# High expression of the putative cancer stem cell marker, DCLK1, in rectal neuroendocrine tumors

**DOI:** 10.3892/ol.2015.3513

**Published:** 2015-07-20

**Authors:** YU IKEZONO, HIRONORI KOGA, MITSUHIKO ABE, JUN AKIBA, AKIHIKO KAWAHARA, TAKAFUMI YOSHIDA, TORU NAKAMURA, HIDEKI IWAMOTO, HIROHISA YANO, MASAYOSHI KAGE, MICHIO SATA, OSAMU TSURUTA, TAKUJI TORIMURA

**Affiliations:** 1 Division of Gastroenterology, Department of Medicine, Kurume University School of Medicine, Kurume, Japan; 2 Research Center for Innovative Cancer Therapy, Kurume University, Kurume, Japan; 3 Department of Pathology, Kurume University School of Medicine, Kurume, Japan; 4 Department of Diagnostic Pathology, Kurume University Hospital, Kurume, Japan

**Keywords:** carcinoids, neuroendocrine tumor, cancer stem cell, NANOG

## Abstract

Doublecortin-like kinase 1 (DCLK1), a microtubule-associated protein, is known to regulate neuronal differentiation, migration and neurogenesis. Recent evidence suggests that the protein is a putative marker for intestinal and pancreatic stem cells, including their cancer stem cell counterparts. The present study conducted immunohistochemical analyses for DCLK1 and the stemness marker, NANOG, in human intestinal neuroendocrine tumors (NETs), as their expression had not been previously investigated in these tumors. Eighteen patients with endoscopically resected rectal NETs were enrolled in the study. The mean age of the patients was 51 years old. The mean diameter of the resected tumors was 5.2 mm, and a histological diagnosis of NET grade G1 was formed for all tumors. Immunohistochemical analysis was performed not only for DCLK1, but also for the known NET markers, synaptophysin, chromogranin A and cluster of differentiation (CD)56. The intensity and distribution of staining were scored on a scale of 0–3 and 0–2, respectively. The sum of the scores was calculated for each specimen. Co-expression of DCLK1 and NANOG was also examined. The mean scores for DCLK1 and synaptophysin were significantly higher than those for chromogranin A (P<0.0001) and CD56 (P<0.01). There were no significant differences in the scores between DCLK1 and synaptophysin or between chromogranin A and CD56. Notably, NANOG was expressed in high quantities in all the tumor tissues studied, showing clear co-expression with DCLK1. In conclusion, DCLK1 may be a novel marker for rectal NET, potentially indicating the presence of the stemness gene product, NANOG.

## Introduction

Neuroendocrine tumors (NETs) originate from cells that produce peptide hormones and amines, and express common neuroendocrine markers, including synaptophysin and chromogranin A. According to the widely accepted concept of the diffuse neuroendocrine system (1), NETs can develop in any organ that has cells with such neuroendocrine signatures. Recently, the incidence of NET has increased worldwide (2,3). The gastrointestinal (GI) tract is the major site of NET development in humans; 56.3% of carcinoid tumors, a former name for NETs, were found in the GI tract in a large-scale NET study analyzing 11,842 patients (4). The clinical course of NETs is diverse; certain NETs are slow growing, like benign tumors, while others behave as aggressive cancers and are associated with a poor prognosis. It was reported that distant metastasis occurred in >70% of NETs when the primary tumor size was >21 mm in diameter, and even 5.5% of NETs if the size was <10 mm (4). Colorectal NETs of >10 mm in diameter showed lymph node metastasis as frequently as colorectal cancers of similar size. Furthermore, a similar trend in prognosis was noted between colorectal NETs and colorectal cancer with lymph node metastasis (5). These findings strongly suggest that colorectal NETs have malignant potential, prompting a reconsideration of the term ‘carcinoid’ (benign carcinoma), which was specified by Oberndorfer more than a century ago (6).

In 2010, NETs, including gastroenteropancreatic NETs, were reclassified into two groups, NET grade G1 and NET grade G2, by the World Health Organization (WHO) (7), with classical carcinoid tumors also classified as NETs. Using this classification, the malignant potential of tumors can be defined by simple evaluation of the Ki-67 positivity index and mitotic count in NET cells. This evaluation is also used for grading rectal NETs, and, to a certain degree, for prognostication (8). However, it remains difficult to predict the malignant potential of tumors prior to treatments, such as surgery and chemotherapy, by using small biopsy samples, as the classification is based on non-specific histopathological markers of cell proliferation. In this context, novel NET-specific markers, such as cancer stem cell (CSC) markers, are required to identify tumor cell origin and behavior.

Doublecortin-like kinase 1 (DCLK1) is a microtubule-associated protein. The function of this protein has been assessed mainly in nerve and neuroblastoma cells, but is not yet fully understood (9–12). Recent accumulating evidence suggests that DCLK1 serves as a putative marker for intestinal and pancreatic stem cells or CSCs, attracting much attention from oncologists and gastroenterologists (13–17).

The present study investigates the expression of DCLK1 in rectal NET tissue, and discusses its relevance to the origin of NET cells and their malignant potential from the point of view of CSCs.

## Patients and methods

### 

#### Patients and tumor tissues

A total of 18 patients with rectal NETs, also known as carcinoid tumors, were enrolled in the present study. Informed consent to participate in the study was obtained from each patient, in accordance with the principles stated in the Declaration of Helsinki and the guidelines of the Ethical Committee of Kurume University (study registration no. 13149). Between 2003 and 2012, the tumors were resected via endoscopic mucosal resection (EMR) at the Kurume University Hospital (Kurume, Japan). In total, 9 patients were male and 9 were female. The mean age of the patients was 51 years old (range, 31–74 years old). The mean longest diameter of the tumors was 5.2 mm (range, 2–8 mm). Histological diagnosis was made by at least two pathologists independently, according to the WHO guidelines for NETs (7). All the resected tumors were diagnosed as G1 grade NETs (Table I). Prior to EMR treatment, no distant metastasis or lymph node metastasis was detected in any of the cases.

#### Immunohistochemical analysis

Expression of DCLK1 and the commonly used neuroendocrine markers, synaptophysin, chromogranin A and CD56, was assessed via immunohistochemical analysis. The intensity of staining was scored on a scale of 0–3 as follows: 0, negative staining; 1, weakly-positive staining; 2, moderately-positive staining; and 3, strongly-positive staining. The signal-positive area was scored on a scale of 0–2 as follows: 0, positive staining in 0–20% of cells; 1, positive staining in 21–60% of cells; and 2, positive staining in 61–100% of cells. The sum of the scores was calculated for each specimen. A total score of ≥2 was judged as positive expression (Table II).

Paraffin-embedded NET tissue samples were cut to prepare 4-µm slices that were mounted on silane-coated glass slides. Immunohistochemical analysis was performed using the primary antibodies listed in Table III. For Ki-67 staining, the BenchMark ULTRA autostainer (Ventana Automated Systems, Inc., Tucson, AZ, USA) was used. Briefly, each slide was heat-treated using CC1 retrieval solution (Ventana Automated Systems, Inc.) for 60 min, and incubated with the antibodies for 30 min. This automated system used the streptavidin-biotin complex method with 3,3′-diaminobenzidine (DAB) as a chromogen (Ventana UltraVIEW DAB detection kit; Ventana Automated Systems, Inc.). Immunostaining for chromogranin A, synaptophysin and CD56 was performed using the similar fully-automated Bond-Max system (Leica Microsystems, Newcastle, UK), with onboard heat-induced antigen retrieval for 10 min, and the Bond Polymer Refine Detection kit (Leica Microsystems). For staining of DCLK1 and NANOG, antigen retrieval was performed by autoclaving tissue sections in 10 mM citrate buffer (pH 6.0) at 120°C for 5 min. The sections were pre-blocked using Protein Block Serum-Free (DakoCytomation, Glostrup, Denmark) and incubated with primary antibodies. Subsequent to being washed, the sections were incubated with EnVision secondary antibodies labeled with horseradish peroxidase-polymer complexes (DakoCytomation) and visualized with DAB. The cell nuclei were counterstained with hematoxylin. Specimens incubated with rabbit IgG alone were used as negative controls.

#### Statistical analysis

Statistical significance was assessed using the Mann-Whitney U test, using StatView 5.0 J software (SAS Institute Inc., Cary, NC, USA). P<0.05 was considered to indicate a statistically significant difference.

## Results

### 

#### Expression of DCLK1 and known neuroendocrine markers

**Figure 1.**
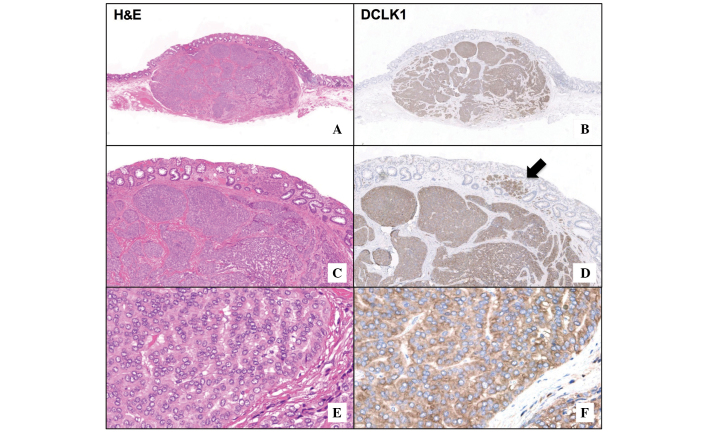
Expression of doublecortin-like kinase 1 (DCLK1) in rectal neuroendocrine tumors (NETs). Endoscopic mucosal resection-treated NET sections (case 4) were stained with (A, C and E) hematoxylin and eosin (H&E) and (B, D and F) anti-DCLK1 antibodies. Note the strong staining and cytoplasmic distribution of immunoreactive DCLK1. (D) The invasive front of the tumor (arrow) is clearly marked by the presence of DCLK1-positive cells in the mucosal layer. Original magnification, (A and B) ×2, (C and D) ×5 and (E and F) ×40.

**Figure 2.**
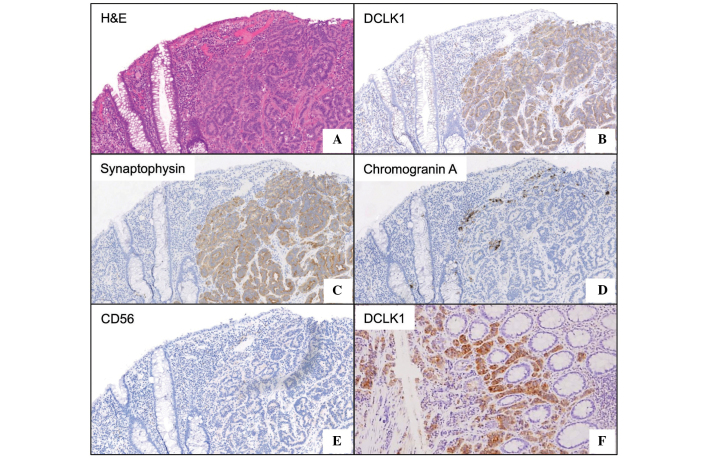
Expression of doublecortin-like kinase 1 (DCLK1) and known neuroendocrine markers. Rectal neuroendocrine tumor (NET) sections (case 13) were stained with (A) hematoxylin and eosin (H&E), and (B) anti-DCLK1, (C) anti-synaptophysin, (D) anti-chromogranin A and (E) anti-cluster of differentiation (CD)56 antibodies. Note the similar levels of strong DCLK1 and synaptophysin expression. The staining score was 5 for each specimen. The score for chromogranin A and CD56 was 3. (F) In a biopsy sample of a rectal polypoid lesion, DCLK1-positive NET cells are clearly observed in periglandular spaces. Original magnification, ×10.

The expression of DCLK1 in rectal NET tissues is demonstrated in Fig. 1. Diffuse cytoplasmic expression of DCLK1 was observed in the tumor area (Fig. 1B and F), while no positive signal was visible in the surrounding non-tumorous tissue. The invasive front of the tumor was clearly marked by the presence of DCLK1-positive cells in the mucosal layer (Fig. 1D). Serial sections of NETs were immunostained with the commonly used NET markers, synaptophysin, chromogranin A and CD56 (Fig. 2).

#### Analysis of immunostaining scores

**Figure 3.**
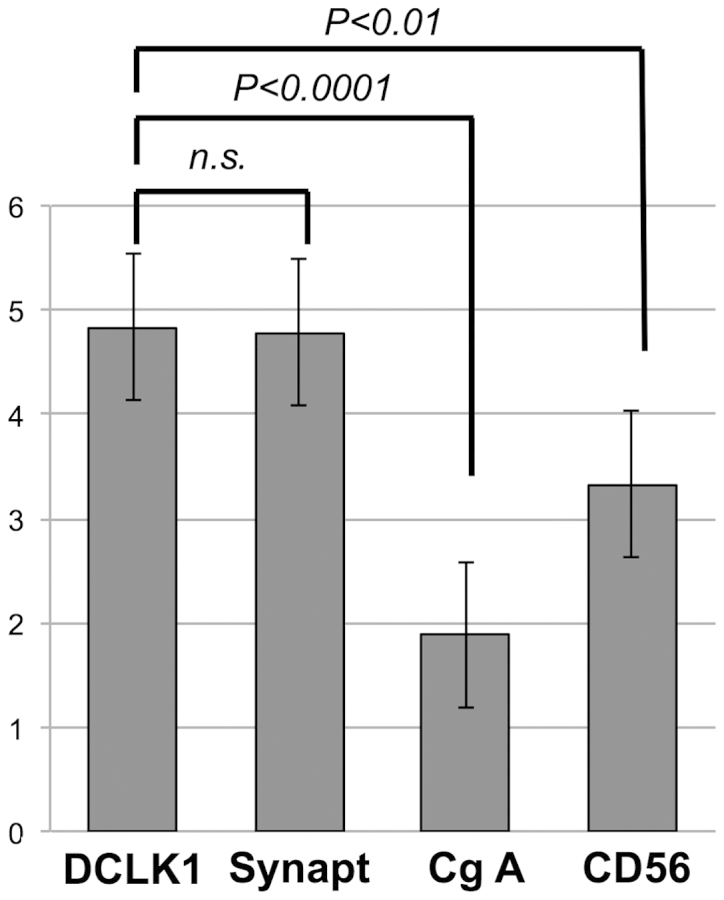
Mean immunostaining scores. The mean ± standard error of the total immunostaining score for doublecortin-like kinase 1 (DCLK1), synaptophysin (Synapt), chromogranin A (CgA) and cluster of differentiation (CD)56 is 4.83±0.12, 4.78±0.15, 1.89±0.54 and 3.33±0.42, respectively, according to the Mann-Whitney U test.

The immunostaining scores, including Ki-67 indices, are summarized in Table IV. DCLK1 protein expression was observed in all the specimens, and the mean ± standard error of the total scores for DCLK1 was 4.83±0.12, which was equivalent to that of synaptophysin (4.78±0.15). These two scores were significantly higher than that of chromogranin A (1.89±0.54; P<0.0001) and CD56 (3.33±0.42; P<0.01) (Fig. 3). There were no significant differences in the scores between DCLK1 and synaptophysin, or between chromogranin A and CD56.

#### Expression of NANOG

**Figure 4.**
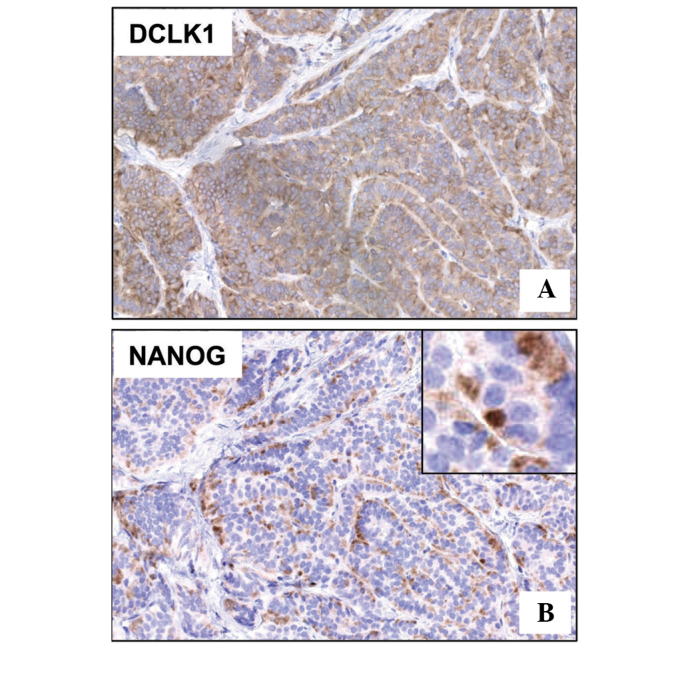
Expression of NANOG in rectal neuroendocrine tumor (NET) tissue. NANOG is expressed in doublecortin-like kinase 1 (DCLK1)-positive rectal NET tissue. Note that the nuclear and cytoplasmic expression of NANOG (inset) is localized at the basal side of trabecular-like structures in the tumor. Original magnification, ×20.

The NANOG stem cell marker was widely expressed in all NET tissues studied (Fig. 4). Nuclear and cytoplasmic expression was observed (Fig. 4), and staining was particularly predominant at the basal side of trabecular-like structures in the tumor.

## Discussion

In the present study, the strong expression of DCLK1 in rectal NETs was demonstrated. DCLK1 has two N-terminal doublecortin domains that bind microtubules and regulate their polymerization (18). The C-terminal serine/threonine protein kinase domain, which has substantial homology to the Ca^2+^/calmodulin-dependent protein kinase, regulates a calcium-signaling pathway, thereby controlling neurogenesis, neuronal migration and apoptosis in the developing brain (9,10). This functional protein is also abundantly expressed in neuronal neoplasms, including neuroblastoma, and confers drug resistance to neoplastic cells (19). On the basis of these findings, DCLK1 was believed to play a fundamental role in tumor cells, including NET cells, where neuroendocrine markers are positive-for promoting cell migration, proliferation and tumorigenesis. Several previous studies have underlined the crucial involvement of DCLK1 in NET cell behavior. In animal models with xenografted colon and pancreatic tumors, the silencing of the *Dclk1* gene resulted in a decrease in tumor size (15,17,20). Although the precise tumor-promoting mechanism of DCLK1 has yet to be fully elucidated, the DCLK1-mediated downregulation of the expression of tumor-suppressing microRNAs (miR), such as miR-145, miR-200 and let-7a, has been demonstrated to be a possible mechanism (17,20). Another proposed mechanism involves the DCLK1-dependent induction of vascular endothelial growth factor receptor and epithelial-mesenchymal transition (EMT)-related factors in tumor cells (17,20). These molecules, involved in the inhibition of tumor suppressors, angiogenesis and EMT, may contribute to the aggressive characteristics of not only cancer cells, but also NET cells.

The present study also investigated the diagnostic value of DCLK1 in NETs. NETs are conventionally diagnosed using hematoxylin-eosin staining and immunostaining for chromogranin A, synaptophysin and CD56. The results showed 100% positivity for synaptophysin, and 83.3 and 44.4% positivity for CD56 and chromogranin A, respectively. These findings are consistent with the findings of a previous study of 114 cases of NETs, which demonstrated 97.4% positivity for synaptophysin, 75.4% for CD56, and 43.0% for chromogranin A (21). Similar to the results for synaptophysin, DCLK1 positivity was also observed in 100% of the tumors in the present study, suggesting its diagnostic value in NETs. It was also noteworthy that DCLK1 staining could detect NET cells in a small rectal polypoid biopsy sample with high sensitivity. The use of DCLK1 immunostaining may extend diagnostic options prior to the treatment of NETs.

CSCs exhibit EMT, which is partly regulated by DCLK1. Recent evidence suggested that the CSC-related stemness gene products, including CDX2, OCT4 and SOX2, were expressed in NETs (22,23). Due to this, the present study assessed whether the expression of DCLK1 correlated with that of NANOG, another marker of stem cells and CSCs. NANOG was found to be highly expressed in the rectal NET tissues, and its distribution of expression overlapped with that of DCLK1. This finding provided insights into the CSC-like nature of NET cells, and it supports previous findings that the knockdown of DCLK1 expression completely blocked the expression of the stemness markers, NANOG, KLF4, OCT4 and SOX2, in human pancreatic cancer cells (20). Although further studies are required, we speculate that the CSC marker, DCLK1, along with stemness gene products, is also involved in the development of NETs, possibly owing to interactions among them under specific conditions. From a therapeutic point of view, targeting DCLK1 using emerging small molecules may open novel avenues for the treatment of cancer, including NETs, in various organs (24,25).

The histological grade of all resected NETs in the present study was G1, possibly as the tumors were small enough to be treatable via EMR. Despite the early developmental stage of the tumors, DCLK1 and NANOG expression was strong and widely distributed throughout. This point is important when considering the origin and malignant potential of NETs. It is known that tuft cells in intestinal crypts are DCLK1-positive and quiescent, and have stem cell-like characteristics (26). Therefore, it is speculated that DCLK1-positive tuft lineage cells are multipotent, or at least bipotent, ectopically transforming into NET cells and orthotopically into intestinal cancer cells, under specific microenvironmental conditions. Recently, tuft cells have been shown to become powerful colon cancer-initiating cells owing to inflammatory insult (27). Therefore, intestinal inflammation may affect the fate of tuft cell transformation.

This study provides original findings and speculates on novel concepts regarding the expression of the CSC and/or tuft cell marker, DCLK1, in NETs. However, as the number of tumor samples and the tumor grade were limited, further large-scale studies are required to determine the role of DCLK1 in NETs more precisely.

## Figures and Tables

**Table I.** Characteristics of the patients and tumors.

Characteristics	Value
Gender	
Male	9
Female	9
Mean age (range), years	51 (31–74)
Tumor grade, n	
G1	18
G2	0
Longest tumor diameter (range), mm	5.4 (2.0–8.0)

**Table II.** Evaluation criteria for immunostaining.

	Score			
	
Criteria	0	1	2	3
Signal intensity	Negative	Mild	Moderate	Strong
Positive area, %	0–10	11–60	61–100	-

**Table III.** Characteristics of antibodies used in this study.

Antigen	Clone/type	Dilution	Antigen retrieval	Source
DCLK1	EPR6085	1:700	H	Epitomics, Burlingame, CA, USA
Synaptophysin	Z66	1:1	H	Invitrogen, Frederick, MD, USA
Chromogranin A	DAK-A3	1:400	H	DakoCytomation, Glostrup, Denmark
CD56	1B6	1:200	H	Leica Microsystems, Newcastle, UK
Ki-67	MIB-1	1:200	H	DakoCytomation, Glostrup, Denmark

DCLK1, doublecortin-like kinase 1; H, heat-activated; CD, cluster of differentiation.

**Table IV.** Immunoreactivity scores for DCLK1 and known neuroendocrine markers.

Case no.	Age, years	Gender	Tumor size, mm	Ki-67 index	DCLK1	Synaptophysin	Chromogranin A	CD56
1	63	F	8×8	<2%	5	5	0	5
2	51	M	3×3	<2%	5	5	5	5
3	65	F	8×8	<2%	3	5	5	5
4	62	F	6×6	<2%	5	5	0	4
5	50	F	5×4	<2%	5	5	5	3
6	39	F	5×5	<2%	5	5	0	3
7	73	M	7×6	<2%	5	5	0	0
8	55	F	3×2	<2%	5	5	0	3
9	46	F	6×6	<2%	5	5	0	5
10	31	F	5×4	<2%	5	5	0	3
11	46	M	7×6	<2%	5	5	0	3
12	56	M	5×4	<2%	5	5	5	5
13	33	M	5×5	<2%	5	5	3	3
14	60	M	3×2	<2%	5	5	0	3
15	34	F	4×3	<2%	5	3	3	0
16	41	M	6×6	<2%	5	3	3	5
17	74	M	2×2	<2%	5	5	5	0
18	30	M	6×6	<2%	4	5	0	5

DCLK1, doublecortin-like kinase 1; M, male, F, female; CD, cluster of differentiation.
